# Human Parvovirus 4 Infection, Cameroon

**DOI:** 10.3201/eid1804.110628

**Published:** 2012-04

**Authors:** Myriam Lavoie, Colin P. Sharp, Jacques Pépin, Christopher Pennington, Yacouba Foupouapouognigni, Oliver G. Pybus, Richard Njouom, Peter Simmonds

**Affiliations:** Université de Sherbrooke, Sherbrooke, Quebec, Canada (M. Lavoie, J. Pépin);; University of Edinburgh, Edinburgh, Scotland, UK (C.P. Sharp, C. Pennington, P. Simmonds);; Centre Pasteur du Cameroun, Yaoundé, Cameroon (Y. Foupouapouognigni, R. Njouom);; University of Oxford, Oxford, UK (O. Pybus)

**Keywords:** Cameroon, human parvovirus 4, PARV4, human partetravirus, epidemiology, viruses

## Abstract

In a post hoc analysis of samples collected in 2009, we determined seroprevalence of parvovirus 4 (PARV4) among elderly Cameroonians. PARV4 seropositivity was associated with receipt of intravenous antimalarial drugs, intramuscular streptomycin, or an intramuscular contraceptive, but not hepatitis C virus seropositivity. Findings suggest parenteral acquisition of some PARV4 infections.

Human parvovirus 4 (PARV4), also known as partetravirus, was identified in 2005 from the plasma of an intravenous drug user (IDU) (*1*). In separate studies that used PCR, PARV4 was subsequently documented in autopsy tissues from IDUs and persons with hemophilia; in bone marrow aspirates from patients with AIDS; and in the blood of transplant recipients, hemodialysis patients, and infants in Ghana (*2**–**5*).

In 2007, 199 (32.4%) of 626 adults tested in Burkina Faso, Democratic Republic of the Congo, and Cameroon were seropositive by first-generation serologic assay for PARV4 (*6*). In South Africa, prevalence was 36% among HIV-infected blood donors but only 4% among their HIV-seronegative counterparts (*6*). Although PARV4 presence in IDUs and hemophilia patients suggests parenteral transmission (*7**,**8*), this route has not yet been studied and other modes of transmission have not been ruled out. The pathogenicity of PARV4 remains unclear, but PARV4 DNA recently was found in the cerebrospinal fluid of 2 children from India who had unexplained encephalitis (*9*).

During 2010, to investigate the epidemiology of PARV4 in Africa, we tested for PARV4 antibodies in serum samples collected during a 2009 study of a defined population of elderly Cameroonians among whom prevalence of hepatitis C virus (HCV) infection was high. Previous exposures to parenteral and sexual risk factors had been documented for this population (*10**–**12*), indicating that this population had been excessively exposed to improperly sterilized syringes and needles and that the main risk factor for HCV was the administration of intravenous antimalarial drugs, mostly before 1960.

## The Study

The ethics committees of the Cameroonian Ministry of Health and the Centre Hospitalier Universitaire de Sherbrooke (Sherbrooke, Quebec, Canada) approved the 2009 study and 2010 follow-up specimen testing. The study was conducted in Ebolowa, southern Cameroon (*10*). Inclusion criteria were age >60 years and consent. Exclusion criteria were dementia or inability to communicate. With cooperation from community leaders, we visited a convenience sample of houses to identify participants. We obtained venous samples from participants and gathered sociodemographic data and information about past intravenous treatment for any disease, past parenteral treatment for infectious diseases, transfusions, scarifications, and circumcision. Vaccine scars were documented.

We performed PARV4 IgG detection on each sample in replicate by indirect ELISA by using baculovirus-expressed viral protein 2 and control antigens (*8*); arbitrary unit (AU) values were calculated relative to a control sample. Because of a high background reactivity observed for this cohort, we additionally stipulated that for positive samples, the optical density ratio (ODR) of viral protein 2 to control must be >1.2; ODRs below this threshold were considered negative.

Serologic assays for HCV and treponemal antibodies were described in the original study by Pepin et al. (*10*). We detected antibodies against hepatitis B core antigen (HBcAg) by using AxSYM (Abbott, Montreal, Quebec, Canada) and analyzed data by using Stata 10.0 (StataCorp LP, College Station, TX, USA). Proportions were compared by using either the χ^2^ or Fisher exact test. Variables associated with PARV4 seropositivity in univariate analysis were tested in logistic regression models through nonautomated forward selection, continuing until no other variable reached significance. Each variable was then eliminated to assess its effect by using likelihood ratio tests. We retained in the final model variables that enhanced the fit at the p<0.05 level.

The study comprised 451 persons 60–102 years of age (median 70 years); 56% were HCV seropositive, 74% had antibodies against *Treponema* (10), and 95% were anti-HBcAg seropositive. Seventy-nine (17.5%) persons carried PARV4 antibodies.

PARV4 antibodies were more prevalent among persons 60–64 years of age than among older persons (Table 1). Prevalence did not vary by sex or by presence of anti-HCV, anti-HBcAg, or treponemal antibodies. The prevalence of anti-PARV4 increased, but not significantly, with exposure to intravenous treatments in general. Receipt of intravenous antimalarial drugs was associated with PARV4 seropositivity, which was also more frequent among persons treated for tuberculosis and among the few women who had received injections of the contraceptive Depo-Provera (Pharmacia & Upjohn Company, New York, NY, USA). PARV4 seropositivity was not associated with treatments delivered by injection against yaws, syphilis, leprosy, or trypanosomiasis (data not shown) or with sexually transmitted infections. PARV4 seropositivity was less common among persons who had a vaccine scar on the left arm.

**Table 1:** Prevalence of human parvovirus 4 by patient characteristics, Cameroon, 2009*

Characteristic	No. virus positive/ no. tested (%)	p value
Age, y		0.04
60–64	32/125 (26)	
65–69	13/96 (14)	
70–74	17/103 (17)	
>75	17/127 (13)	
Sex		0.15
M	25/178 (14)	
F	54/273 (20)	
HCV serologic results		0.61
Negative	29/178 (16)	
Positive	47/252 (19)	
Anti-HBcAg		1.00
Negative	3/21 (14)	
Positive	76/430 (18)	
Treponemal antibodies		0.06
Absent	28/119 (24)	
Present	51/332 (15)	
Intravenous treatment for malaria		0.04
No	29/216 (13)	
Yes	50/235 (21)	
Intravenous treatment for other diseases		0.93
No	42/239 (18)	
Yes	37/212 (17)	
No. past intravenous treatments		0.38
0	12/88 (14)	
1–3	33/206 (16)	
>4	25/116 (22)	
Unknown	9/41 (22)	
Tuberculosis		0.04
No	72/433 (17)	
Yes, treated with oral drugs only	4/12 (33)	
Yes, treatment included streptomycin	3/6 (50)	
Transfusion		0.09
No	76/408 (19)	
Yes	3/43 (7)	
Depo-Provera injections†		0.006
No	50/268 (19)	
Yes	4/5 (80)	
Scarifications		0.88
No	30/165 (18)	
Yes	49/286 (17)	
Vaccine scar, left arm		0.005
Absent	17/53 (32)	
Present	61/397 (15)	
Vaccine scar, right arm		0.76
Absent	27/165 (16)	
Present	51/284 (18)	
Circumcision (males only)		0.74
Medical	9/73 (12)	
Traditional	16/105 (15)	

*HCV, hepatitis C virus, HBcAg, hepatitis B core antigen. †Pharmacia & Upjohn Company, New York, NY, USA.

In multivariate analysis (Table 2), PARV4 seropositivity was associated with younger age, intravenous receipt of antimalarial drugs, and parenteral receipt of antituberculosis treatment (the latter was of borderline significance) and was less common among persons with a left-sided vaccine scar. In that model, Depo-Provera injections were associated with PARV-4 seropositivity among women (adjusted odds ratio 17.27, 95% CI 1.57–189.78; p = 0.02).

**Table 2:** Correlates of study participants and human parvovirus 4 infection in multivariate analysis, Cameroon, 2009

Participant characteristic	All participants			After exclusions*	
	Adjusted odds ratio (95% CI)	p value		Adjusted odds ratio (95% CI)	p value
Age group, y					
60–64	2.21 (1.13–4.31)	0.02		2.88 (1.16–7.17)	0.02
65–69	1.01 (0.46–2.24)	0.98		1.20 (0.39–3.70)	0.76
70–74	1.16 (0.54–2.46)	0.71		1.51 (0.55–4.16)	0.42
>75	1.00			1.00	
Tuberculosis					
No	1.00			1.00	
Yes, treated with oral drugs only	2.09 (0.58–7.54)	0.26		2.91 (0.63–13.51)	0.17
Yes, treatment included streptomycin	5.21 (0.99–27.37)	0.05		20.96 (1.67–262.99)	0.02
Vaccine scar, left arm					
Absent	1.00			1.00	
Present	0.37 (0.19–0.71)	0.003		0.32 (0.13–0.78)	0.01
Intravenous treatment for malaria					0.06
No	1.00			1.00	
Yes	1.92 (1.13–3.24)	0.015		1.98 (0.97–4.03)	0.06

*After exclusion of 81 participants with borderline negative results and 35 with borderline positive results.

To confirm that associations were not biased by assay sensitivity, we conducted a secondary analysis that excluded 81 borderline PARV4-negative persons (AU >0.5 and ODR <1.2) and 35 borderline PARV4-positive persons (AU 0.5–2.0, ODR >1.2) (Table 2). The same factors as in the main analysis were associated with PARV4 seropositivity; receipt of intravenous antimalarial drugs was not significant in the smaller sample.

## Conclusions

We retrospectively analyzed samples obtained during a study of elderly Cameroonians from an area where HCV infection was hyperendemic and in which we had collected much information about potential parenteral modes of transmission of blood-borne viruses but less information about other routes (*10*). Because this was a cross-sectional study, the time sequence of exposure routes and PARV4 infection could not be determined. Thus, our results should be considered exploratory.

The sensitivity, specificity, and ability of our assay to identify seroconversions are comparable to those of PCR-based methods for determining active infections and past exposure (*7**–**9**,**13*). Exclusion of samples showing low antibody levels that might represent nonspecific reactivity had little effect on the analysis of risk factors.

The results provide some evidence for parenteral transmission of PARV4 in the study community. As was HCV infection (*10*), PARV4 infection was associated with receipt of intravenous antimalarial therapy. This risk factor was found for half of the population we studied, whereas intramuscular Depo-Provera and streptomycin were administered to few patients. In univariate analysis, PARV4 seropositivity was also more common in patients treated with oral antituberculosis drugs. Although the seroprevalence of PARV4 increased with past exposure to intravenous treatments in general, this finding was not statistically significant because antibodies against PARV4 were common among persons who reported no such treatments. This finding, and the lack of association between PARV4 and HCV seropositivity, suggests that other, nonparenteral modes of transmission existed.

PARV4 seropositivity was more common in persons 60–64 years of age than in older persons. This finding has 3 potential explanations. First, exposure to the virus might have fluctuated over time. Second, titers of antibodies against PARV4 might progressively wane, eventually leading to false negative results. Third, PARV4 infection might increase long-term risk for death, although this explanation seems unlikely.

Absence of a vaccine scar on the left arm was associated with PARV4 seropositivity. Historical and epidemiologic data suggest that in Cameroon, the left side was used for smallpox vaccine and the right side for *Mycobacterium bovis* BCG (*14**,**15*). Failure of scar development after smallpox vaccination might reflect immunologic characteristics associated with greater susceptibility to PARV4 infection.

Our findings suggest that some parenteral transmission of PARV4 occurred among elderly Cameroonians, but parenteral transmission might not have been the main route of infection. The association with past tuberculosis, although perhaps coincidental, is intriguing and deserves further study.

## References

[1] Jones MS, Kapoor A, Lukashov VV, Simmonds P, Hecht F, Delwart E. DNA viruses identified in patients with acute viral infection syndrome. J Virol. 2005;79:8230–6. 10.1128/JVI.79.13.8230-8236.2005 15956568 PMC1143717

[2] Longhi E, Bestetti G, Acquaviva V, Foschi A, Piolini R, Meroni L, Human parvovirus 4 in the bone marrow of Italian patients with AIDS. AIDS. 2007;21:1481–3. 10.1097/QAD.0b013e3281e38558 17589196

[3] Vallerini D, Barozzi P, Quadrelli C, Bosco F, Potenza L, Riva G, Parvoviruses in blood donors and transplant patients, Italy. Emerg Infect Dis. 2008;14:185–6. 10.3201/eid1401.070610 18258108 PMC2600167

[4] Touinssi M, Reynaud-Gaubert M, Gomez C, Thomas P, Dussol B, Berland Y, Parvovirus 4 in French in-patients: a study of hemodialysis and lung transplant cohorts. J Med Virol. 2011;83:717–20. 10.1002/jmv.22003 21328388

[5] Panning M, Kobbe R, Vollbach S, Drexler JF, Adjei S, Adjei O, Novel human parvovirus 4 genotype 3 in infants, Ghana. Emerg Infect Dis. 2010;16:1143–6. 10.3201/eid1607.100025 20587191 PMC3321913

[6] Sharp CP, Vermeulen M, Nébié Y, Djoko CF, LeBreton M, Tamoufe U, Epidemiology of human parvovirus 4 infection in sub-Saharan Africa. Emerg Infect Dis. 2010;16:1605–7. 20875290 10.3201/eid1610.101001 PMC3294412

[7] Simmonds P, Manning A, Kenneil R, Carnie FW, Bell JE. Parenteral transmission of the novel human parvovirus PARV4. Emerg Infect Dis. 2007;13:1386–8. 18252117 10.3201/eid1309.070428 PMC2857296

[8] Sharp CP, Lail A, Donfield S, Simmons R, Leen C, Klenerman P, High frequencies of exposure to the novel human parvovirus, PARV4 in haemophiliacs and injecting drug users detected by a serological assay for PARV4 antibodies. J Infect Dis. 2009;200:1119–25. 10.1086/605646 19691429 PMC2914696

[9] Benjamin LA, Lewthwaite P, Vasanthapuram R, Zhao G, Sharp C, Simmonds P, Human parvovirus 4 (PARV4) as potential cause of encephalitis in children, India. Emerg Infect Dis. 2011;17:1484–7. 21801629 10.3201/eid1708.110165 PMC3381555

[10] Pépin J, Lavoie M, Pybus OG, Pouillot R, Foupouapouognigni Y, Rousset D, HCV transmission during medical interventions and traditional practices in colonial Cameroon: potential implications for the emergence of HIV-1. Clin Infect Dis. 2010;51:768–76. 20735242 10.1086/656233

[11] Njouom R, Nerrienet E, Dubois M, Lachenal G, Rousset D, Vessière A, The hepatitis C virus epidemic in Cameroon: genetic evidence for rapid transmission between 1920 and 1960. Infect Genet Evol. 2007;7:361–7. 10.1016/j.meegid.2006.10.003 17137845

[12] Pépin J, Labbé AC. Noble goals, unforeseen consequences: the control of tropical diseases in colonial central Africa and the iatrogenic transmission of blood-borne viruses. Trop Med Int Health. 2008;13:744–53. 10.1111/j.1365-3156.2008.02060.x 18397182

[13] Lahtinen A, Kivela P, Hedman L, Kumar A, Kantele A, Lappalainen M, Serodiagnosis of primary infections with human parvovirus 4, Finland. Emerg Infect Dis. 2011;17:79–82. 10.3201/eid1701.100750 21192859 PMC3204632

[14] Blanchard M. Précis d’épidémiologie. Médecine préventive et hygiène coloniales. Paris: Vigot Frères; 1938.

[15] Brunel M. La tuberculose pulmonaire au Cameroun en 1958, endémie tuberculeuse, formes cliniques, traitement, prophylaxie. Bull Soc Pathol Exot. 1958;51:920–35. 13662803

